# Initial validation of a multi-station non-contrast dark blood approach for the diagnosis of peripheral arterial disease

**DOI:** 10.1186/1532-429X-11-S1-P24

**Published:** 2009-01-28

**Authors:** Georgeta Mihai, YiuCho Chung, Mbabazi Kariisa, Orlando P Simonetti, Sanjay Rajagopalan

**Affiliations:** 1 The Ohio State University, Columbus, OH USA; 2 Siemens Healthcare USA, Inc, Columbus, OH USA

**Keywords:** Magnetic Resonance Angiographic, Peripheral Arterial Disease, Superficial Femoral Artery, Intermittent Claudication, Lumen Area

## Introduction

Current magnetic resonance angiographic (MRA) diagnosis of peripheral arterial disease (PAD) is based on contrast-enhanced angiography (ce-MRA), a luminographic technique lacking information on atherosclerotic plaque burden. Moreover, the recent links between nephrogenic systemic fibrosis disease and gadolinium based contrasts limits use of this technique in PAD patients with impaired renal function.

## Purpose

This study aims to evaluate the feasibility and accuracy of a multistationT1w-SPACE [1] dark blood MRA sequence to assess disease in the aorto-iliac and superficial femoral artery (SFA) (inflow vessels) by comparing it to a multistation ce-MRA protocol with identical spatial resolution.

## Methods

### Sequences

T1w-SPACE [1] is a highly efficient 3D TSE technique due to its long echo train and very short echo spacing. Blood signal is suppressed in T1w-SPACE by dephasing due to flow in the readout direction. Both T1w-SPACE and ce-MRA were acquired with isotropic 1 mm resolution. Imaging parameters are listed in Table 1.

**Table 1:** Acquisition parameters for T1w-SPACE and ce-MRA

T1w-SPACE	ECG trg.	TR/TE (ms)	Echo train	Slices	Scan time (s)	BW (Hz/pixel)	iPAT	NEX
Abd. Aorta	Yes	RR/25	180	80	14:30–22:30	587	2	3.5
Thighs (SFA)	No	700/23	72	60	11:30	587	2	1.4
**ce-MRA**	**ECG trg**.	**TR/TE (ms)**	**Echo train**	**Slices**	**Scan time (s)**	**BW (Hz/pixel)**	**iPAT**	**NEX**
Abd. aorta	NA	3/1	NA	88	0:14	445	3	NA
SFA/Lower legs	NA	3/1	NA	66	0:15	445	2	NA

### Imaging

The study was approved by the Institutional Review Board. Two normal volunteers and six patients (with normal kidney function) diagnosed with PAD and symptoms of intermittent claudication were included in the study. Imaging was performed on a 1.5 T system (MAGNETOM Avanto, Siemens, Erlangen) using two body matrix coils for the abdominal/pelvic area and a peripheral coil for thighs and lower legs. The imaging protocol included localizers, coronal acquisition of abdominal aorta and SFA using T1w-SPACE, followed by ce-MRA covering the abdomen, thighs and the lower legs.

### Analysis

T1w-SPACE and ce-MRA acquisitions for each subject were co-registered (abdominal aorta, and then SFA) on a workstation (FUSION, Siemens Healthcare, Inc.). Three, 1 mm multiplanar images (with 3 mm gap) perpendicular to the arteries were created at each of the following locations: celiac, superior mesenteric, renal, halfway between renal and bifurcation, iliac bifurcation, and at both right and left iliac, internal and external iliac, proximal, medial and distal SFA levels. Two experienced observers measured lumen area at each of these 17 locations on both T1w-SPACE and ce-MRA images, as well as the wall thickness area in T1w-SPACE for plaque burden quantification.

## Results

**Figure 1:**
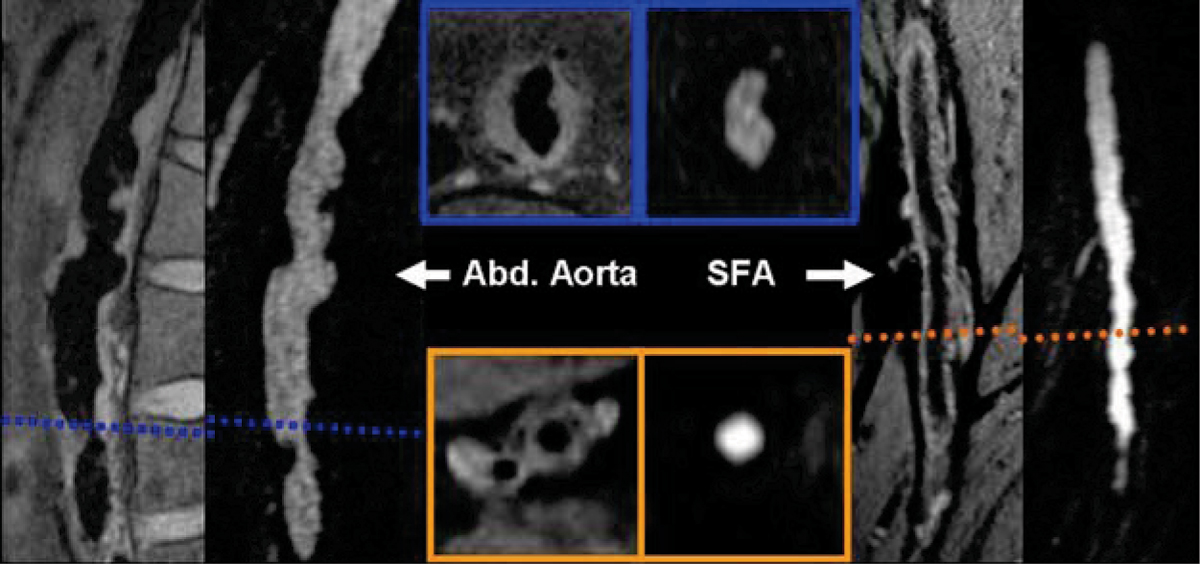
**T1w-SPACE (left) and ce-MRA (right) images of segments of abdominal aorta (left group) and SFA (right group) showing the dark and bright lumen, as well as the arterosclerotic plaque in T1w-Space (left middle group)**.

**Figure 2:**
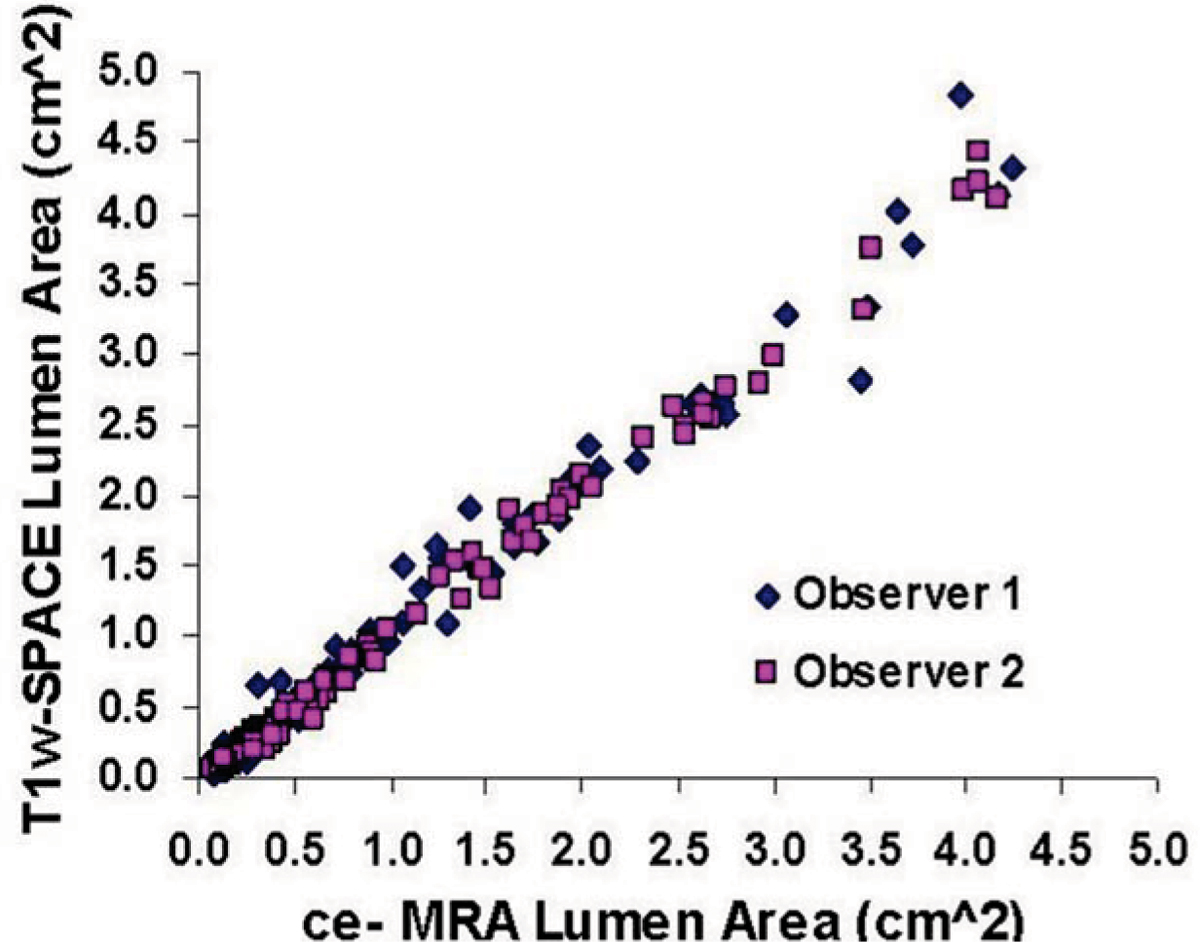
**Graph showing the strong correlation between T1w-SPACE and ce-MRA lumen area, and strong inter-observer agreement**.

Figure 1 shows an example of T1w-SPACE versus ce-MRA images depicting the lumen area and the vessel wall atherosclerotic plaque. Quantitative comparison of lumen areas with ce-MRA and T1w-SPACE revealed strong correlation between the two techniques (r > 0.9) and strong inter-observer agreement between the two imaging methods (see Figure 2).

## Conclusion

T1w SPACE imaging of inflow vessels (aorto-iliac and SFA) is feasible and an attractive alternative to ce-MRA in patients with PAD. The ability to assess atherosclerostic plaque and vascular remodeling is an additional significant improvement over other non-contrast [2] and ce-MRA techniques.
